# 微小残留病和IKZF1缺失在预测成人急性B淋巴细胞白血病患者预后中的价值

**DOI:** 10.3760/cma.j.cn121090-20231002-00157

**Published:** 2024-03

**Authors:** 诗雨 邓, 家旺 区, 子聪 黄, 俊杰 陈, 梓红 蔡, 启发 刘, 红升 周

**Affiliations:** 南方医科大学南方医院血液科，广州 510515 Department of Hematology, Nanfang Hospital, Southern Medical University, Guangzhou 510515, China

**Keywords:** 基因，IKZF1, 白血病，B淋巴细胞，急性, 微小残留病, 儿童特点化疗方案, Gene, IKZF1, Leukemia, B-cell, acute, Minimal residual disease, Pediatric-inspired regimen therapy

## Abstract

**目的:**

评估微小残留病（MRD）和IKZF1基因缺失在接受儿童特点化疗方案的成人急性B淋巴细胞白血病 (B-ALL) 中的预后价值。

**方法:**

回顾性分析2016年1月至2020年9月南方医科大学南方医院收治的149例成人B-ALL患者的预后情况。采用Cox回归模型进行预后因素分析。

**结果:**

149例患者完全缓解（CR）率为93.2％，5年总生存（OS）率和累积复发率（CIR）分别为（54.3±5.0）％和（47.5±5.2）％。Cox回归分析发现诱导治疗后第45天的微小残留病（MRD_3_）阳性与患者复发风险独立相关（*HR*＝2.535，95％*CI* 1.122～5.728，*P*＝0.025），IKZF1基因缺失与患者的死亡风险独立相关（*HR*＝1.869，95％*CI* 1.034～3.379，*P*＝0.039）。基于MRD_3_和IKZF1基因状态，我们将149例成人B-ALL患者分为高危组［MRD_3_阳性和（或）IKZF1基因缺失］和低危组（MRD_3_阴性且IKZF1基因野生型）。两组的5年OS率分别为（45.5±6.0）％和（69.4±8.6）％（*P*<0.001），5年CIR分别为（61.6±8.3）％和（25.5±6.5）％（*P*<0.001），差异均有统计学意义。多因素分析表明，高危组是影响OS（*HR*＝3.937，95％*CI* 1.975～7.850，*P*<0.001）以及CIR（*HR*＝4.037，95％*CI* 2.095～7.778，*P*<0.001）的独立危险因素。

**结论:**

MRD结合IKZF1的预后分层系统可更有效地预测成人B-ALL患者的临床结局，有助于指导患者治疗方案的选择。

## Abstract (en)

**Objective:**

To reassess the prognostic value of minimal residual disease（MRD）and IKZF1 gene deletions in adults with B-cell acute lymphoblastic leukemia（B-ALL）who received pediatric-specific chemotherapy regimens during the Nanfang Hospital PDT-ALL-2016 trial.

**Methods:**

We retrospectively analyzed the prognosis of 149 adult patients with B-ALL who were admitted to Nanfang Hospital from January 2016 to September 2020. Prognostic factors were identified using Cox regression models.

**Results:**

The complete remission rate was 93.2％ in 149 patients, with a 5-year overall survival（OS）rate of（54.3±5.0）％ and a cumulative incidence of relapse（CIR）of（47.5±5.2）％. The Cox regression analysis revealed that MRD positivity at day 45（MRD_3_）after induction therapy was independently associated with relapse risk（*HR*＝2.535, 95％ *CI* 1.122–5.728, *P*＝0.025）. Deletion of IKZF1 gene was independently associated with mortality risk（*HR*＝1.869, 95％ *CI* 1.034–3.379, *P*＝0.039）. Based on MRD_3_ and IKZF1 gene status, we categorized adult patients with B-ALL into the low-risk（MRD_3_-negative and IKZF1 gene deletion-negative）and high-risk（MRD_3_-positive and/or IKZF1 gene wild type）groups. The 5-year OS and CIR rates were（45.5±6.0）％ *vs*（69.4±8.6）％（*P*<0.001）and（61.6±8.3）％ *vs*（25.5±6.5）％（*P*<0.001）, respectively, in the high-risk and low-risk groups, respectively. The multivariate analysis showed that the high-risk group was an independent risk factor for OS（*HR*＝3.937, 95％ *CI* 1.975–7.850, *P*<0.001）and CIR（*HR*＝4.037, 95％ *CI* 2.095–7.778, *P*<0.001）.

**Conclusion:**

The combined use of MRD and IKZF1 gene in prognostic stratification can improve clinical outcome prediction in adult patients with B-ALL, helping to guide their treatment.

随着化疗方案的变化以及预后的显著改善，传统的急性淋巴细胞白血病（ALL）高危因素及细胞遗传学预后分组是否仍能有效预测患者预后仍未可知[1]–[4]。除基线分层外，微小残留病（MRD）是儿童和成人ALL患者最重要的预后因素，且不受方法学、阈值以及测量时间点影响[5]–[8]。然而，MRD本身不足以完全预测预后[9]–[11]。体细胞遗传学异常定义了不同的生物学亚群，不同亚群具有不同的潜在预后标志物。

IKZF1基因的编码蛋白Ikaros是血液淋巴系统发育必需的转录因子，负责调控淋巴细胞分化发育。Ikaros蛋白有6个锌指结构域，其中氨基末端的4个锌指结构介导DNA结合。据报道，在B细胞前体（BCP）ALL中，编码氨基末端锌指结构的IKZF1外显子缺失发生率较高，且与不良预后有关[12]–[17]。

目前已有研究显示MRD与细胞遗传学异常的组合可以重新对ALL患者进行预后分层，但将MRD与IKZF1基因缺失结合的研究尚不足[10],[11],[18]–[24]。特异性遗传学异常在多大程度上影响ALL早期的治疗反应尚不清楚，对于整合遗传学异常和MRD来对患者进行分层的最佳方法也没有共识。因此我们在儿童样方案PDT-ALL-2016研究队列中探讨MRD与IKZF1对B-ALL患者的预后意义。

## 病例与方法

1. 病例：回顾性分析149例于2016年1月至2020年9月在南方医科大学南方医院确诊的成人B-ALL患者的临床资料。患者的诊断分型采用WHO 2016标准。

2. 治疗：所有患者均接受PDT-ALL-2016方案进行治疗，该方案是一种以GRAALL 2003方案为基础、培门冬酶强化、联合抗代谢药物的儿童特点化疗方案。所有患者先接受1周的地塞米松的预治疗，后予以VICLP（长春新碱、去甲氧柔红霉素、培门冬酶、泼尼松）方案和CAM（环磷酰胺、阿糖胞苷和6-巯基嘌呤）方案诱导缓解。Ph染色体阳性者加用酪氨酸激酶抑制剂（TKI，达沙替尼）。诱导缓解后予阿糖胞苷、甲氨蝶呤、环磷酰胺联合培门冬酶巩固化疗[25]。根据患者诊断时的危险因素以及治疗后早期反应进行危险分层，高危组进入allo-HSCT路径。该研究于2016年获得南方医院机构审查委员会批准（批件号：NFEC-2018-002），并遵循《赫尔辛基宣言》。

3. MRD检测：诱导化疗后，采用流式细胞术（FCM）动态监测MRD。MRD检测的时间点为诱导化疗后第14天（MRD_1_）、第24天（MRD_2_）、第45天（MRD_3_）。3个时间点MRD的阈值分别为1％、0.1％、0.01％。当该时间点检测的MRD>阈值时定义为MRD阳性，≤阈值为MRD阴性[25]。

4. IKZF1基因缺失检测：方法学上采用断点特异性多重PCR和多重连接探针法检测IKZF1基因缺失。从诊断性骨髓样品中分离单个核细胞，采用QIAamp DNA Micro Kit试剂盒提取基因组DNA，并用NanoDrop ND-100分析DNA质量。参考Caye等[26]的研究，我们分别在IKZF1基因第2、4、7、8号外显子上设计基因组上、下游引物。扩增反应参考ABI 7500定量PCR仪使用说明书设置反应程序、采集荧光信号、自动设置Ct值。软件中出现标准扩增曲线，Ct值<36定义为IKZF1基因缺失，余者为IKZF1基因野生型[17]。

5. 随访及预后评估：随访采用查阅门诊病历、住院病历或电话方式。随访截止日期为2022年5月6日。总生存（OS）期从确诊之日起计算，直至死亡或末次随访。累积复发率（CIR）的计算自完全缓解之日起至任何部位复发为止，并将非复发死亡率（NRM）视为竞争事件。无上述事件发生者计算至末次随访日。

6. 统计学处理：统计数据采用频率、中位数等进行描述。计数资料组间比较采用Fisher精确检验或卡方检验。采用Kaplan-Meier法分析OS和CIR，采用Log-rank检验（双侧）比较组间差异。采用Cox回归模型估计风险比及其95％ *CI*。数据分析采用SPSS 25.0软件。*P*<0.05为差异具有统计学意义。绘图采用R软件。

## 结果

1. 患者临床资料：共149例患者纳入研究。男77例（51.7％），女72例（48.3％）。中位年龄为29（18～81）岁；≥35岁者65例（43.6％）。中位随访时间为34个月。其中，76例复发，70例死亡。根据供者可及性及患者个人意愿，74例（49.6％）患者接受allo-HSCT。

2. IKZF1基因缺失：149例患者中，60例（40.3％）存在IKZF1基因缺失。其中，1个、2个及3个外显子缺失的患者分别为46例（76.7％）、11例（18.3％）、3例（5.0％）。4～7号外显子缺失最为常见（38例，63.0％）。IKZF1基因缺失与较高的MRD_1_阳性率（48.3％对27.0％，*P*＝0.008）、Ph染色体阳性率（61.7％对34.8％，*P*＝0.001）相关（表1）。生存分析结果表明，IKZF1基因缺失组5年OS率明显低于IKZF1基因野生型组（40.4％对61.3％，*P*＝0.014）（图1A），5年CIR明显高于IKZF1基因野生型组（52.6％对40.8％，*P*＝0.020）（图1B）。Cox回归单因素和多因素分析结果表明，IKZF1基因缺失是影响患者OS（*HR*＝1.869，95％*CI* 1.034～3.379，*P*＝0.039）的独立危险因素（表2、3）。

**表1:** 成人急性B淋巴细胞白血病患者IKZF1缺失组和野生型组的临床特征比较

特征	IKZF1野生型（89例）	IKZF1缺失（60例）	χ^2^值	*P*值
性别（例，男/女）	46/43	31/29	<0.001	0.998
年龄>35岁[例（%）]	38（42.7）	27（45.0）	0.077	0.781
WBC≥30×10^9^/L[例（%）]	32(36.0)	29（48.3）	2.271	0.132
LDH≥600 IU/L[例（%）]	33(37.1)	20（33.3）	0.219	0.640
免疫分型pro-B[例（%）]	10（11.2）	7（11.7）	0.007	0.935
MRD_1_>1%[例（%）]	24（27.0）	29（48.3）	7.140	0.008
MRD_2_>0.1%[例（%）]	20（22.5）	22（36.7）	3.567	0.059
MRD_3_>0.01%[例（%）]	26（29.2）	24（40.0）	1.870	0.171
Ph染色体阳性[例（%）]	31（34.8）	37（61.7）	10.403	0.001
首疗程完全缓解［例（%）］	85（95.5）	54（90.0）	1.735	0.188
移植［例（％）］	42（47.2）	32（53.3）	0.541	0.462
死亡［例（％）］	28（31.5）	30（50.0）	5.181	0.023
复发［例（%）］	32（36.0）	31（51.7）	3.625	0.057

注 MRD_1_、MRD_2_、MRD_3_分别为诱导化疗后第14天、第24天、第45天微小残留病水平

**图1:**
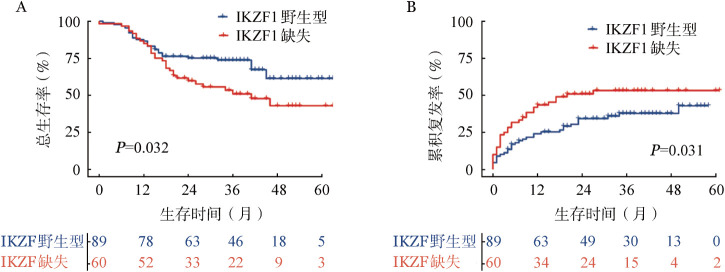
IKZF1基因缺失组与IKZF1基因野生型组成人急性B淋巴细胞白血病患者总生存（A）及累积复发（B）曲线

**表2:** 成人急性B淋巴细胞白血病患者预后的单因素分析

因素	总生存			累积复发率		
	*HR*	95％*CI*	*P*值	*HR*	95％*CI*	*P*值
性别（男，女）	1.252	0.747~2.098	0.394	1.200	0.732~1.968	0.470
年龄>35岁	1.841	1.096~3.091	0.021	1.388	0.845~2.279	0.196
WBC≥30×10^9^/L	1.670	0.997~2.799	0.051	1.928	1.174~3.167	0.009*
LDH≥600 IU/L	1.105	0.645~1.893	0.717	0.934	0.552~1.580	0.798
免疫分型（pro-B,其他）	1.760	0.703~4.411	0.228	1.526	0.657~3.546	0.326
MRD_1_>1%	1.342	0.779~2.312	0.289	1.641	0.978~2.752	0.061
MRD_2_>0.1%	1.951	1.129~3.372	0.017	2.423	1.437~4.085	0.001
MRD_3_>0.01%	2.118	1.235~3.632	0.006	2.768	1.648~4.649	<0.001
Ph染色体阳性	1.567	0.934~2.631	0.089	1.354	0.825~2.221	0.230
IKZF1基因缺失	1.736	1.036~2.909	0.036	1.703	1.038~2.793	0.035
移植	0.330	0.187~0.582	<0.001	0.463	0.277~0.774	0.003

注 MRD_1_、MRD_2_、MRD_3_分别为诱导化疗后第14天、第24天、第45天微小残留病水平

**表3:** 成人急性B淋巴细胞白血病患者预后的多因素分析

因素	*HR*	95％*CI*	*P*值
总生存			
IKZF1基因缺失	1.869	1.034~3.379	0.039
移植	0.330	0.174~0.626	0.001
累积复发率			
WBC≥30×10^9^/L	1.911	1.074~3.402	0.028
MRD_3_>0.01%	2.535	1.122~5.728	0.025
移植	0.429	0.240~0.768	0.004

注 MRD_3_：诱导化疗第45天微小残留病水平

3. MRD：生存分析表明，MRD_1_阳性组的5年OS率和CIR与MRD_1_阴性组差异无统计学意义。与MRD_2_阴性组相比，MRD_2_阳性组患者的5年OS率较低［（39.6±9.3）％对（60.8±6.2）％，*P*＝0.012］，5年CIR较高［（79.8±14.8）％对（37.1±5.3）％，*P*<0.001］，差异均有统计学意义。与MRD_3_阴性组相比，MRD_3_阳性组患者的5年OS率较低［（40.7±8.0）％对（62.4±6.7）％，*P*<0.001］，5年CIR较高［（75.8±11.0）％对（33.2±5.4）％，*P*<0.001］（图2）。MRD_3_阴性与首疗程完全缓解率有关（100.0％ 对 86.0％，*P*＝0.001）（表4）。Cox回归单因素和多因素分析结果表明，MRD_3_阳性是影响患者CIR（*HR*＝2.535，95％*CI* 1.122～5.728，*P*＝0.025）的独立危险因素（表2、3）。

**图2:**
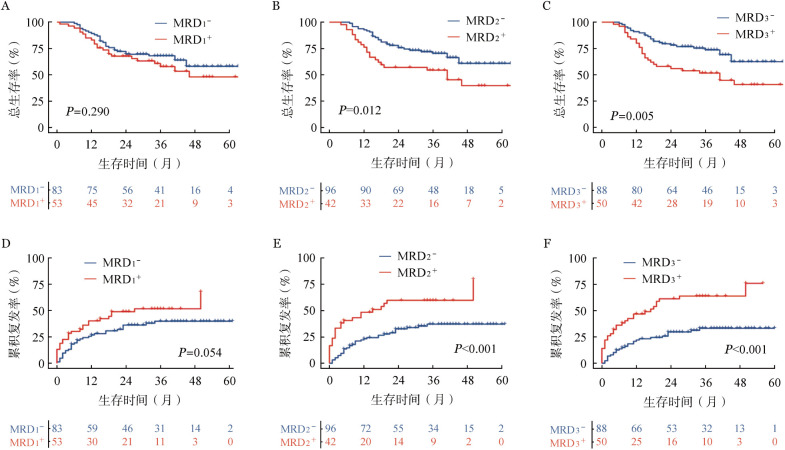
微小残留病（MRD）动态变化对成人急性B淋巴细胞白血病患者总生存率（A、B、C）及累积复发率（D、E、F）的影响 注 MRD_1_、MRD_2_、MRD_3_分别为诱导化疗后第14天、第24天、第45天微小残留病水平

**表4:** 成人急性B淋巴细胞白血病患者MRD_3_阳性组和阴性组的临床特征比较

特征	MRD_3_阴性（88例）	MRD_3_阳性（50例）	统计量	*P*值
性别（例，男/女）	44（50.0）	23（46.0）	0.204	0.651
年龄>35岁[（%）]	37（42.0）	22（44.0）	0.050	0.823
WBC≥30×10^9^/L[例（%）]	34（38.6）	20（40.0）	0.025	0.875
LDH≥600 IU/L[例（%）]	16（18.2）	13（26.0）	1.174	0.279
免疫分型pro-B[例（%）]	10（11.4）	6（12.0）	0.013	0.911
Ph染色体阳性［例（％）］	44（50.0）	19（38.0）	1.851	0.174
IKZF1基因缺失［例（％）］	30（34.1）	24（48.0）	2.590	0.108
首疗程完全缓解［例（%）］	88（100）	43（86.0）	10.234	0.001
移植［例（％）］	44（50.0）	26（52.0）	0.051	0.821
死亡［例（％）］	26（29.5）	27（54.0）	8.060	0.005
复发［例（%）］	27（30.7）	31（62.0）	12.835	<0.001

注 MRD_3_指在诱导治疗后第45天的微小残留病水平。缓解率采用Fisher精确检验，其余变量采用卡方检验

4. 新的危险分层：基于以上结果，我们结合MRD及IKZF1基因缺失，将成人B-ALL队列分为4组：MRD^−^ IKZF1野生型、MRD^+^IKZF1野生型、MRD^−^IKZF1缺失、MRD^+^IKZF1缺失。生存分析提示MRD^−^IKZF1野生型组预后最好，其余三组预后较差，但组间差异无统计学意义（图3A、3B）。因此，我们将MRD^−^IKZF1野生型组定义为低危（LR）组，剩余3组合并为高危（HR）组。HR组的5年OS率［（45.5±6.0）％对（69.4±8.6）％，*P*<0.001；图3C）及5年CIR［（61.6±8.3）％对（25.5±6.5）％，*P*<0.001；图3D］明显劣于LR组。多因素分析表明，危险分层HR是影响患者OS（*HR*＝3.937，95％*CI* 1.975～7.850，*P*< 0.001）以及CIR（*HR*＝4.037，95％*CI* 2.095～7.778，*P*<0.001）的独立不良预后因素（表5），这一结果表明，新的分层系统可有效预测B-ALL患者的临床结局。

**图3:**
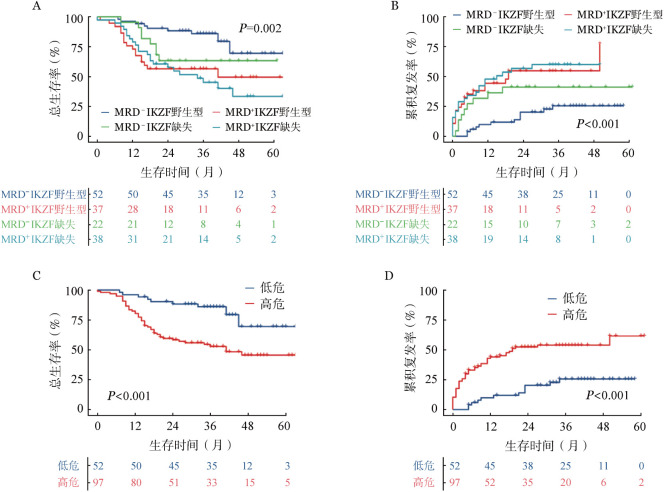
诱导化疗后第45天的微小残留病（MRD_3_）和IKZF1基因缺失对成人急性B淋巴细胞白血病患者总生存及累积复发率的影响 A、C 根据MRD_3_和IKZF1基因缺失分组对总生存的影响；B、D 根据MRD_3_和IKZF1基因缺失分组对累积复发率的影响 注 低危组：MRD_3_阴性且IKZF1基因野生型；高危组：MRD_3_阳性和（或）IKZF1基因缺失

**表5:** 新分类的风险组对成人急性B淋巴细胞白血病患者预后影响的多因素分析

因素	总生存			累积复发率		
	*HR*	95％*CI*	*P*值	*HR*	95％*CI*	*P*值
性别（男，女）	1.194	0.683~2.086	0.534	1.196	0.700~2.043	0.512
年龄>35岁	1.497	0.848~2.645	0.164	1.248	0.727~2.143	0.422
WBC≥30×10^9^/L	1.478	0.812~2.692	0.202	2.030	1.166~3.535	0.012
LDH≥600 IU/L	1.276	0.734~2.218	0.388	0.918	0.530~1.588	0.759
免疫分型（pro-B,其他）	2.025	0.774~5.298	0.151	1.670	0.689~4.049	0.257
Ph染色体阳性	0.988	0.531~1.839	0.969	0.889	0.503~1.572	0.686
危险分层（低危，高危）	3.937	1.975~7.850	<0.001	4.037	2.095~7.778	<0.001
移植	0.284	0.154~0.521	<0.001	0.378	0.218~0.656	0.001

## 讨论

B-ALL是一组异质性疾病[27]–[28]，因此，采用有效的预后标志物进行分层治疗，对指导患者治疗至关重要。尽管MRD被认为是儿童和成人ALL中最重要的危险因素，但其指导患者分层和基于风险的治疗的预测价值有限，部分早期治疗反应良好的患者仍然面临着复发的风险。转录因子IKZF1（IKAROS）是淋巴细胞分化的重要调节因子。IKZF1的改变在B-ALL的发病机制中发挥着关键作用，并且还与耐药性和复发相关[29]。越来越多证据表明，IKZF1缺失是B-ALL患者的独立不良预后因素[16],[18]–[19]。传统的危险分层策略仅考虑MRD或细胞遗传学异常，然而，目前已有多项研究发现MRD必须在遗传学的背景下解释，才能最大限度地提高其有效性。

2010年Kang等[21]报道了MRD结合基因表达谱可以将处于复发高风险的ALL患儿进一步区分为高、中、低三个风险组。随后Waanders等[10]发现综合使用MRD和IKZF1改变状态可准确预测儿童ALL的复发。2018年Stanulla等[24]在儿童B-ALL中描述了一种新的MRD依赖性的预后极差的高风险组，定义为IKZF1plus。之后，国内外多个研究团队又陆续证明了MRD与基因表达谱结合在成人ALL中也有很高的预后价值[11],[18],[23]。

在本研究中，我们评估了MRD和IKZF1基因缺失在成人B-ALL 患者中的预后价值，并结合这两个因素，为接受儿童特点方案治疗的成人B-ALL患者构建了强有力的新的预后分层。HR组临床结局均劣于LR组。表明新的分层体系对患者的危险事件具有非常强大的预测能力。在Beldjord等[11]的研究中，风险分层仅针对Ph阴性患者，而华西医院2017年的研究报道了MRD结合IKZF1在Ph阳性患者中也有不错的预后分层作用[23]。值得注意的是，在我们的PDT-ALL-2016方案中，大多数Ph阳性白血病患者在确诊后都接受了TKI治疗。这种干预措施可降低复发风险，从而消除Ph阳性的不良预后价值。因而，我们的新的风险分层不仅适用于Ph阴性患者，也适用于接受了TKI治疗的Ph阳性成人B-ALL患者。

鉴于本研究为单中心回顾性研究，因此后续还需进行多中心的大型前瞻性研究以证实本研究所得出的结论。此外，我们缺乏其他基因预后亚组的信息，通过更全面的基因组技术获取更广泛的信息，可以进一步扩展和加强MRD结合基因谱的预后分层。

我们的研究表明，随着儿童特点化疗方案在成人ALL患者中的应用，IKZF1缺失筛查和诱导治疗后MRD检测可能是目前识别成人B-ALL患者复发和死亡风险的最佳工具。因此，通过将两者结合起来能构建有效的风险分层工具，更好地指导治疗。
